# Di-μ-thio­cyanato-bis­[bis­(tri-*p*-tolyl­phosphine)silver(I)] 0.35-hydrate

**DOI:** 10.1107/S1600536810010032

**Published:** 2010-03-27

**Authors:** Nozipho M. Khumalo, Reinout Meijboom, Alfred Muller, Bernard Omondi

**Affiliations:** a Synthesis and Catalysis Research Centre, Department of Chemistry, University of Johannesburg, PO Box 524, Auckland Park, Johannesburg 2006, South Africa

## Abstract

In the binuclear centrosymmetric title compound, [Ag_2_(NCS)_2_(C_21_H_21_P)_4_]·0.35H_2_O, a pseudo-polymorph of [Ag_2_(NCS)_2_(C_21_H_21_P)_4_]·2CH_3_CN, the Ag atom is coordinated by two phosphine ligands and two bridging thio­cyanate ligands in a distorted tetra­hedral configuration. The crystal structure exhibits inter­molecular C—H⋯π inter­actions.

## Related literature

For a general introduction to the coordination chemistry of silver–phosphine complexes, see: Meijboom *et al.* (2009 ▶). For the original preparation of silver–phosphine complexes, see: Mann *et al.* (1937 ▶). For related silver(I)–thio­cyanate complexes, see: Bowmaker *et al.* (1997 ▶); Effendy *et al.* (2005 ▶), Venter *et al.* (2007 ▶), Omondi & Meijboom (2010 ▶). For related silver(I)–tri-*p*-tolyl­phosphine complexes, see: Meijboom *et al.* (2006 ▶); Meijboom (2006 ▶, 2007 ▶); Meijboom & Muller (2006 ▶); Venter *et al.* (2006 ▶). For bond-length data, see: Allen (2002 ▶).
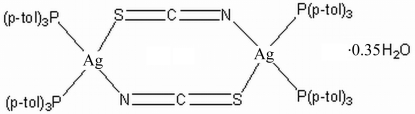


## Experimental

### 

#### Crystal data

[Ag_2_(NCS)_2_(C_21_H_21_P)_4_]·0.35H_2_O
*M* *_r_* = 1554.94
Triclinic, 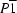

*a* = 10.5470 (6) Å
*b* = 13.5063 (8) Å
*c* = 14.9779 (8) Å
α = 91.575 (1)°
β = 110.064 (1)°
γ = 105.615 (1)°
*V* = 1913.15 (19) Å^3^
*Z* = 1
Mo *K*α radiation
μ = 0.70 mm^−1^
*T* = 173 K
0.42 × 0.35 × 0.15 mm

#### Data collection

Bruker APEXII CCD diffractometer
Absorption correction: multi-scan (*SADABS*; Bruker, 2004 ▶) *T*_min_ = 0.758, *T*_max_ = 0.903
23789 measured reflections
9487 independent reflections
8868 reflections with *I* > 2σ(*I*)
*R*_int_ = 0.022

#### Refinement

*R*[*F* ^2^ > 2σ(*F* ^2^)] = 0.032
*wR*(*F* ^2^) = 0.090
*S* = 0.85
9487 reflections
425 parameters
5 restraints
H-atom parameters constrained
Δρ_max_ = 1.67 e Å^−3^
Δρ_min_ = −1.70 e Å^−3^

### 

Data collection: *APEX2* (Bruker, 2004 ▶); cell refinement: *SAINT-Plus* (Bruker, 2004 ▶); data reduction: *SAINT-Plus*; program(s) used to solve structure: *SHELXS97* (Sheldrick, 2008 ▶); program(s) used to refine structure: *SHELXL97* (Sheldrick, 2008 ▶); molecular graphics: *ORTEP-3 for Windows* (Farrugia, 1997 ▶), *PLATON* (Spek, 2009 ▶) and *DIAMOND* (Brandenburg & Putz, 2005 ▶); software used to prepare material for publication: *WinGX* (Farrugia, 1999 ▶).

## Supplementary Material

Crystal structure: contains datablocks global, I. DOI: 10.1107/S1600536810010032/hg2658sup1.cif

Structure factors: contains datablocks I. DOI: 10.1107/S1600536810010032/hg2658Isup2.hkl

Additional supplementary materials:  crystallographic information; 3D view; checkCIF report

## Figures and Tables

**Table:** 

C—N	1.160 (3)
C—S	1.656 (2)
N—Ag	2.3519 (18)
Ag—P1	2.4516 (5)
Ag—P2	2.4987 (5)
Ag—S^i^	2.6062 (6)

**Table:** 

N—C—S	178.0 (2)
C—N—Ag	142.81 (16)
C—S—Ag^i^	97.85 (7)
N—Ag—P1	115.23 (5)
N—Ag—P2	91.90 (5)
P1—Ag—P2	119.826 (18)
N—Ag—S^i^	105.61 (5)
P1—Ag—S^i^	111.461 (18)
P2—Ag—S^i^	110.655 (19)

**Table:** 

C—N—Ag—S^i^	51.7 (3)

Symmetry code: (i) 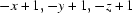.

**Table 2:** Hydrogen-bond geometry (Å, °) *Cg*1 and *Cg*6 are the centroids of the C111–C116 and C231–C236 benzene rings, respectively.

*D*—H⋯*A*	*D*—H	H⋯*A*	*D*⋯*A*	*D*—H⋯*A*
C135—H135⋯*Cg*6^ii^	0.95	2.86	3.772 (2)	161
C225—H225⋯*Cg*1^iii^	0.95	2.73	3.568 (2)	147

Symmetry codes: (ii) 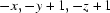; (iii) 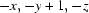.

**Fig. 1.**
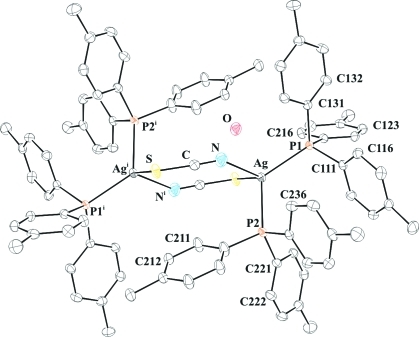
The structure (I), showing 50% probability displacement ellipsoids. H atoms have been omitted for clarity. For the C atoms, the first digit indicates ring number and the second digit indicates the position of the atom in the ring. Primed atoms were generated by symmetry operator (1-x, 1-y, 1-z).

**Fig. 2.**
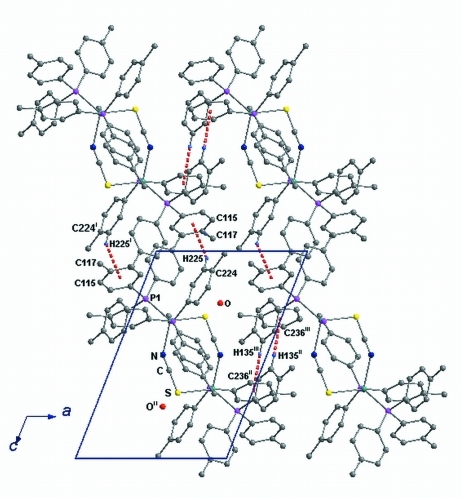
Packing pattern of (I) as viewed down the crystallographic b axis where the C—H···π intermolecular interactions are shown in dashed lines [Symmetry codes: (i) -x, -y+1, -z; (ii) -x+1, -y+1, -z+1 and (iii) x+1, y, z].

## supplementary crystallographic information

### Comment

Silver(I) complexes of the type [AgLnX] (L is a tertiary phosphine or arsine, n = 1–4 and X is a coordinating or noncoordinating anion) were first prepared by Mann *et al.* (1937) and were the first crystallographic examples of metal phosphine complexes. These compounds display a rich diversity of structural types due to the interplay of parameters such as the geometric flexibility of Ag(I), the bite angle, the electronic properties of the group 15 donor ligand, the coordination of the supporting ligand, etc. (Meijboom *et al.*, 2009).

As part of work that was aimed at the identification of roles the above mentioned properties play during the crystallization of simple silver(I) salts with Group 15 donor ligands with initial focus on tri-*p*-tolylphosphine complexes (Meijboom *et al.*, 2006; Meijboom, 2006; Meijboom & Muller, 2006; Venter *et al.*, 2006; Meijboom, 2007), we present here a pseudo-polymorph of the previously reported [Ag_2_(NCS)_2_(C_21_H_21_P)_4_]2CH_3_CN (Venter *et al.*, 2007), [Ag_2_(NCS)_2_(C_21_H_21_P)_4_]0.35H_2_O (I). Complex (I) was left standing on the bench top in the lab for a long period of time during which it supposedly absorbed moisture into its structure.

The asymmetric unit of the title compound, Fig. 1, comprises half a unit of the Ag^I^ complex (the other half generated by the symmetry operator -x+1, -y+1, -z+1) and 0.35 molecule of H_2_O. The bond lengths (Allen *et al.*, 1987) and angles (Table 1) are within the normal ranges and are comparable to those of related complexes such as the pseudo-polymorph [Ag_2_(NCS)_2_(C_21_H_21_P)_4_].2CH_3_CN (Venter *et al.* 2007), [Ag_2_(NCS)_2_{P(4—FC_6_H_4_)_3_}_4_] (Omondi & Meijboom, 2010) and other silver(I) thiocyanate complexes, (Bowmaker *et al.* 1997; Effendy *et al.* 2005).

The geometry around the Ag(I) atom is a slightly distorted tetrahedral which is coordinated by the two SCN anions and two phosphine ligands resulting in a dimeric species in which the two Ag(I) centres are bridged by the SCN anions (Table 1).

The crystal structure is stabilized by pairs of C—H···π intermolecular interactions along the crystallographic a and c axes [H225···Cg1 = 2.73 Å, C225—H225···Cg1 = 147° and H135···Cg6 = 2.86 Å, C135—H135···Cg6 = 161° (Fig. 2). Cg1 and Cg6 are the centroids of the C111/C112/C113/C114/C115/C116 and C231/C232/C233/C234/C235/C236 benzene rings]. The symmetry operator for the two interactions is -x, -y+1, -z+1. The two C—H···π interactions result in dimeric pairs of the adjacent molecules involved.

### Experimental

AgSCN (0.08g, 0.49 mmol) and P(p</>-tol)_3_ (0.30g, 0.98 mmol) were dissolved in warm pyridine to give a clear solution which on cooling and solvent evaporation deposited colourless crystals of [Ag_2_(NCS)_2_(C_21_H_21_P)_4_].H_2_O in good yield.

### Refinement

All hydrogen atoms were positioned geometrically, with C–H = 0.95 Å for aromatic hydrogens and 0.98 Å for methyl hydrogens, and allowed to ride on their parent atoms with U_iso_(H) = 1.2U_eq_(C).

CheckCif Alerts explanations

Applying restraints does not seem to remove Hirshfeld Test Diff. There is partial occupation of the O atom. EADP restaraints were applied. The su's on the Cell Angles are true values. The Solvent Disorder fraction too small.

### Crystal data

**Table:**

[Ag_2_(NCS)_2_(C_21_H_21_P)_4_]·0.35H_2_O	*Z* = 1
*M**_r_* = 1554.94	*F*(000) = 803.2
Triclinic, *P*1	*D*_x_ = 1.35 Mg m^−^^3^
Hall symbol: -P 1	Mo *K*α radiation, λ = 0.71073 Å
*a* = 10.5470 (6) Å	Cell parameters from 19388 reflections
*b* = 13.5063 (8) Å	θ = 1.5–28.4°
*c* = 14.9779 (8) Å	µ = 0.70 mm^−^^1^
α = 91.575 (1)°	*T* = 173 K
β = 110.064 (1)°	Plate, colourless
γ = 105.615 (1)°	0.42 × 0.35 × 0.15 mm
*V* = 1913.15 (19) Å^3^	

### Data collection

**Table:**

Bruker APEXII CCD diffractometer	8868 reflections with *I* > 2σ(*I*)
φ and ω scans	*R*_int_ = 0.022
Absorption correction: multi-scan (*SADABS*; Bruker, 2004)	θ_max_ = 28.4°, θ_min_ = 1.5°
*T*_min_ = 0.758, *T*_max_ = 0.903	*h* = −13→14
23789 measured reflections	*k* = −17→17
9487 independent reflections	*l* = −19→19

### Refinement

**Table:**

Refinement on *F*^2^	5 restraints
Least-squares matrix: full	H-atom parameters constrained
*R*[*F*^2^ > 2σ(*F*^2^)] = 0.032	*w* = 1/[σ^2^(*F*_o_^2^) + (0.0542*P*)^2^ + 4.2409*P*] where *P* = (*F*_o_^2^ + 2*F*_c_^2^)/3
*wR*(*F*^2^) = 0.090	(Δ/σ)_max_ = 0.001
*S* = 0.85	Δρ_max_ = 1.67 e Å^−^^3^
9487 reflections	Δρ_min_ = −1.70 e Å^−^^3^
425 parameters	

### Special details

**Table:**

Geometry. All esds (except the esd in the dihedral angle between two l.s. planes) are estimated using the full covariance matrix. The cell esds are taken into account individually in the estimation of esds in distances, angles and torsion angles; correlations between esds in cell parameters are only used when they are defined by crystal symmetry. An approximate (isotropic) treatment of cell esds is used for estimating esds involving l.s. planes.

### Fractional atomic coordinates and isotropic or equivalent isotropic displacement parameters (Å^2^)

**Table:**

	*x*	*y*	*z*	*U*_iso_*/*U*_eq_	Occ. (<1)
C	0.3928 (2)	0.51437 (16)	0.57467 (14)	0.0177 (4)	
C111	−0.0803 (2)	0.40359 (15)	0.16962 (14)	0.0156 (4)	
C112	−0.0451 (2)	0.47931 (16)	0.11306 (15)	0.0195 (4)	
H112	0.0384	0.4879	0.0993	0.023*	
C113	−0.1307 (2)	0.54193 (16)	0.07693 (16)	0.0209 (4)	
H113	−0.1054	0.5925	0.0383	0.025*	
C114	−0.2536 (2)	0.53172 (16)	0.09658 (15)	0.0201 (4)	
C115	−0.2896 (2)	0.45523 (18)	0.15105 (15)	0.0218 (4)	
H115	−0.3738	0.4462	0.164	0.026*	
C116	−0.2048 (2)	0.39130 (17)	0.18733 (15)	0.0194 (4)	
H116	−0.2319	0.3393	0.2242	0.023*	
C117	−0.3407 (3)	0.60450 (19)	0.06084 (18)	0.0288 (5)	
H11A	−0.4174	0.5912	0.0858	0.043*	
H11B	−0.3807	0.5932	−0.0094	0.043*	
H11C	−0.2805	0.6764	0.0829	0.043*	
C121	0.0701 (2)	0.26637 (15)	0.13329 (14)	0.0161 (4)	
C122	−0.0348 (2)	0.23392 (16)	0.04197 (14)	0.0186 (4)	
H122	−0.1181	0.2548	0.0258	0.022*	
C123	−0.0171 (2)	0.17082 (17)	−0.02548 (15)	0.0223 (4)	
H123	−0.0891	0.1491	−0.0873	0.027*	
C124	0.1038 (2)	0.13926 (16)	−0.00379 (15)	0.0219 (4)	
C125	0.2092 (2)	0.17344 (17)	0.08672 (16)	0.0230 (4)	
H125	0.2933	0.1535	0.1023	0.028*	
C126	0.1929 (2)	0.23641 (17)	0.15464 (15)	0.0203 (4)	
H126	0.2659	0.2592	0.216	0.024*	
C127	0.1228 (3)	0.06905 (19)	−0.07552 (17)	0.0303 (5)	
H12A	0.1933	0.1091	−0.0999	0.045*	
H12B	0.0325	0.0397	−0.1289	0.045*	
H12C	0.1548	0.0128	−0.0442	0.045*	
C131	−0.0335 (2)	0.23911 (15)	0.28655 (14)	0.0156 (4)	
C132	−0.1165 (2)	0.14053 (16)	0.23791 (15)	0.0204 (4)	
H132	−0.1356	0.1259	0.1714	0.024*	
C133	−0.1715 (2)	0.06369 (16)	0.28579 (16)	0.0224 (4)	
H133	−0.2278	−0.0028	0.2514	0.027*	
C134	−0.1454 (2)	0.08243 (16)	0.38360 (16)	0.0203 (4)	
C135	−0.0651 (2)	0.18157 (17)	0.43150 (15)	0.0231 (4)	
H135	−0.0486	0.1967	0.4976	0.028*	
C136	−0.0086 (2)	0.25880 (16)	0.38437 (15)	0.0205 (4)	
H136	0.0472	0.3254	0.4188	0.025*	
C137	−0.1997 (3)	−0.00233 (18)	0.43636 (18)	0.0270 (5)	
H13A	−0.294	−0.0456	0.3949	0.04*	
H13B	−0.205	0.0286	0.4944	0.04*	
H13C	−0.1354	−0.0451	0.4541	0.04*	
C211	0.4186 (2)	0.74299 (19)	0.44628 (18)	0.0284 (3)	
C212	0.5508 (2)	0.72876 (19)	0.48426 (18)	0.0284 (3)	
H212	0.5692	0.6724	0.4568	0.034*	
C213	0.6573 (2)	0.79579 (17)	0.56212 (16)	0.0215 (4)	
H213	0.7479	0.7852	0.5862	0.026*	
C214	0.6338 (2)	0.87726 (17)	0.60492 (15)	0.0223 (4)	
C215	0.4993 (2)	0.88878 (19)	0.56982 (18)	0.0284 (3)	
H215	0.4799	0.9429	0.5997	0.034*	
C216	0.3917 (3)	0.82230 (19)	0.49124 (18)	0.0284 (3)	
H216	0.3001	0.8313	0.4685	0.034*	
C217	0.7509 (3)	0.95257 (19)	0.68683 (17)	0.0298 (5)	
H21A	0.7794	1.0196	0.6646	0.045*	
H21B	0.832	0.9254	0.7101	0.045*	
H21C	0.7172	0.9616	0.739	0.045*	
C221	0.3423 (2)	0.71958 (16)	0.24398 (15)	0.0178 (4)	
C222	0.3987 (2)	0.82711 (16)	0.24913 (16)	0.0221 (4)	
H222	0.4115	0.8715	0.3038	0.026*	
C223	0.4360 (2)	0.86902 (17)	0.17507 (18)	0.0254 (4)	
H223	0.4738	0.9421	0.1797	0.03*	
C224	0.4194 (2)	0.80636 (18)	0.09378 (17)	0.0233 (4)	
C225	0.3644 (2)	0.69922 (17)	0.08922 (16)	0.0210 (4)	
H225	0.3523	0.6549	0.0347	0.025*	
C226	0.3273 (2)	0.65665 (16)	0.16311 (15)	0.0182 (4)	
H226	0.291	0.5835	0.1587	0.022*	
C227	0.4579 (3)	0.8511 (2)	0.0122 (2)	0.0364 (6)	
H22A	0.3754	0.8648	−0.0348	0.055*	
H22B	0.4882	0.8017	−0.0185	0.055*	
H22C	0.535	0.9161	0.037	0.055*	
C231	0.1245 (2)	0.68002 (15)	0.32687 (14)	0.0171 (4)	
C232	0.0794 (2)	0.76033 (17)	0.28263 (16)	0.0210 (4)	
H232	0.1369	0.8067	0.2555	0.025*	
C233	−0.0494 (2)	0.77291 (17)	0.27792 (16)	0.0229 (4)	
H233	−0.0778	0.8288	0.2485	0.028*	
C234	−0.1373 (2)	0.70543 (17)	0.31532 (15)	0.0203 (4)	
C235	−0.0920 (2)	0.62480 (17)	0.35919 (16)	0.0223 (4)	
H235	−0.1502	0.5777	0.3853	0.027*	
C236	0.0369 (2)	0.61247 (16)	0.36516 (15)	0.0207 (4)	
H236	0.066	0.5574	0.3957	0.025*	
C237	−0.2777 (2)	0.7183 (2)	0.30916 (19)	0.0288 (5)	
H23A	−0.27	0.7475	0.3721	0.043*	
H23B	−0.35	0.6506	0.2894	0.043*	
H23C	−0.3044	0.7651	0.2621	0.043*	
N	0.3107 (2)	0.47534 (14)	0.49960 (13)	0.0211 (3)	
S	0.50745 (5)	0.56667 (5)	0.68345 (4)	0.02352 (11)	
Ag	0.270935 (15)	0.468455 (11)	0.334821 (10)	0.01632 (5)	
P1	0.05204 (5)	0.34022 (4)	0.22977 (3)	0.01412 (10)	
P2	0.29159 (5)	0.65734 (4)	0.33849 (4)	0.01642 (10)	
O	0.5565 (9)	0.2031 (7)	0.2512 (6)	0.0211 (3)	0.176 (4)

### Atomic displacement parameters (Å^2^)

**Table:**

	*U*^11^	*U*^22^	*U*^33^	*U*^12^	*U*^13^	*U*^23^
C	0.0168 (8)	0.0207 (9)	0.0169 (7)	0.0042 (7)	0.0089 (6)	0.0024 (7)
C111	0.0150 (8)	0.0153 (9)	0.0141 (8)	0.0037 (7)	0.0032 (7)	−0.0010 (7)
C112	0.0170 (9)	0.0190 (9)	0.0220 (10)	0.0041 (7)	0.0073 (8)	0.0036 (8)
C113	0.0211 (10)	0.0167 (9)	0.0219 (10)	0.0031 (7)	0.0058 (8)	0.0048 (7)
C114	0.0200 (9)	0.0182 (9)	0.0182 (9)	0.0074 (8)	0.0014 (8)	−0.0024 (7)
C115	0.0187 (9)	0.0292 (11)	0.0191 (9)	0.0094 (8)	0.0071 (8)	0.0007 (8)
C116	0.0186 (9)	0.0226 (10)	0.0171 (9)	0.0056 (8)	0.0069 (8)	0.0040 (7)
C117	0.0273 (11)	0.0261 (11)	0.0314 (12)	0.0150 (9)	0.0032 (9)	0.0020 (9)
C121	0.0167 (9)	0.0156 (8)	0.0154 (9)	0.0034 (7)	0.0064 (7)	0.0009 (7)
C122	0.0200 (9)	0.0187 (9)	0.0153 (9)	0.0050 (7)	0.0048 (7)	0.0017 (7)
C123	0.0284 (11)	0.0206 (10)	0.0139 (9)	0.0041 (8)	0.0053 (8)	−0.0001 (7)
C124	0.0310 (11)	0.0165 (9)	0.0196 (10)	0.0037 (8)	0.0135 (9)	0.0015 (7)
C125	0.0224 (10)	0.0226 (10)	0.0259 (11)	0.0071 (8)	0.0112 (9)	0.0001 (8)
C126	0.0177 (9)	0.0216 (10)	0.0188 (9)	0.0041 (8)	0.0046 (8)	−0.0017 (8)
C127	0.0455 (14)	0.0276 (12)	0.0223 (11)	0.0124 (10)	0.0170 (10)	−0.0010 (9)
C131	0.0154 (8)	0.0156 (9)	0.0154 (9)	0.0045 (7)	0.0051 (7)	0.0017 (7)
C132	0.0229 (10)	0.0175 (9)	0.0185 (9)	0.0021 (8)	0.0081 (8)	−0.0011 (7)
C133	0.0248 (10)	0.0155 (9)	0.0253 (10)	0.0019 (8)	0.0107 (9)	−0.0007 (8)
C134	0.0202 (10)	0.0196 (9)	0.0232 (10)	0.0063 (8)	0.0100 (8)	0.0056 (8)
C135	0.0270 (11)	0.0229 (10)	0.0168 (9)	0.0036 (8)	0.0079 (8)	0.0008 (8)
C136	0.0234 (10)	0.0171 (9)	0.0167 (9)	0.0015 (8)	0.0059 (8)	−0.0006 (7)
C137	0.0323 (12)	0.0228 (10)	0.0300 (11)	0.0066 (9)	0.0172 (10)	0.0089 (9)
C211	0.0235 (6)	0.0269 (6)	0.0285 (6)	0.0104 (4)	0.0006 (4)	−0.0100 (4)
C212	0.0235 (6)	0.0269 (6)	0.0285 (6)	0.0104 (4)	0.0006 (4)	−0.0100 (4)
C213	0.0157 (9)	0.0235 (10)	0.0219 (10)	0.0043 (8)	0.0040 (8)	0.0012 (8)
C214	0.0220 (10)	0.0180 (9)	0.0198 (10)	0.0035 (8)	0.0011 (8)	0.0003 (8)
C215	0.0235 (6)	0.0269 (6)	0.0285 (6)	0.0104 (4)	0.0006 (4)	−0.0100 (4)
C216	0.0235 (6)	0.0269 (6)	0.0285 (6)	0.0104 (4)	0.0006 (4)	−0.0100 (4)
C217	0.0292 (12)	0.0235 (11)	0.0245 (11)	0.0036 (9)	−0.0015 (9)	−0.0037 (9)
C221	0.0143 (9)	0.0165 (9)	0.0207 (9)	0.0033 (7)	0.0052 (7)	0.0007 (7)
C222	0.0203 (10)	0.0163 (9)	0.0267 (11)	0.0026 (8)	0.0075 (8)	−0.0009 (8)
C223	0.0206 (10)	0.0180 (10)	0.0342 (12)	0.0013 (8)	0.0091 (9)	0.0048 (9)
C224	0.0164 (9)	0.0272 (11)	0.0260 (11)	0.0055 (8)	0.0078 (8)	0.0087 (9)
C225	0.0173 (9)	0.0243 (10)	0.0205 (10)	0.0065 (8)	0.0058 (8)	0.0012 (8)
C226	0.0151 (9)	0.0171 (9)	0.0197 (9)	0.0034 (7)	0.0043 (7)	0.0004 (7)
C227	0.0356 (14)	0.0398 (14)	0.0342 (13)	0.0050 (11)	0.0173 (11)	0.0149 (11)
C231	0.0157 (9)	0.0165 (9)	0.0153 (9)	0.0024 (7)	0.0031 (7)	−0.0022 (7)
C232	0.0193 (10)	0.0196 (10)	0.0230 (10)	0.0041 (8)	0.0075 (8)	0.0047 (8)
C233	0.0227 (10)	0.0222 (10)	0.0232 (10)	0.0082 (8)	0.0062 (8)	0.0049 (8)
C234	0.0184 (9)	0.0214 (10)	0.0178 (9)	0.0027 (8)	0.0055 (8)	−0.0037 (7)
C235	0.0234 (10)	0.0196 (10)	0.0248 (10)	0.0038 (8)	0.0120 (8)	0.0016 (8)
C236	0.0237 (10)	0.0179 (9)	0.0219 (10)	0.0054 (8)	0.0105 (8)	0.0028 (8)
C237	0.0226 (11)	0.0303 (12)	0.0359 (13)	0.0099 (9)	0.0121 (10)	0.0022 (10)
N	0.0218 (8)	0.0235 (9)	0.0175 (6)	0.0056 (7)	0.0077 (6)	0.0034 (6)
S	0.0158 (2)	0.0346 (3)	0.0168 (2)	0.0025 (2)	0.00627 (18)	−0.0048 (2)
Ag	0.01599 (8)	0.01542 (8)	0.01422 (8)	0.00279 (5)	0.00302 (5)	−0.00025 (5)
P1	0.0136 (2)	0.0137 (2)	0.0132 (2)	0.00289 (17)	0.00369 (17)	0.00016 (17)
P2	0.0160 (2)	0.0139 (2)	0.0169 (2)	0.00268 (18)	0.00463 (18)	−0.00128 (17)
O	0.0218 (8)	0.0235 (9)	0.0175 (6)	0.0056 (7)	0.0077 (6)	0.0034 (6)

### Geometric parameters (Å, °)

**Table:**

C—N	1.160 (3)	C211—P2	1.822 (2)
C—S	1.656 (2)	C212—C213	1.392 (3)
C111—C116	1.395 (3)	C212—H212	0.95
C111—C112	1.400 (3)	C213—C214	1.379 (3)
C111—P1	1.820 (2)	C213—H213	0.95
C112—C113	1.387 (3)	C214—C215	1.387 (3)
C112—H112	0.95	C214—C217	1.510 (3)
C113—C114	1.397 (3)	C215—C216	1.399 (3)
C113—H113	0.95	C215—H215	0.95
C114—C115	1.387 (3)	C216—H216	0.95
C114—C117	1.508 (3)	C217—H21A	0.98
C115—C116	1.396 (3)	C217—H21B	0.98
C115—H115	0.95	C217—H21C	0.98
C116—H116	0.95	C221—C226	1.398 (3)
C117—H11A	0.98	C221—C222	1.402 (3)
C117—H11B	0.98	C221—P2	1.827 (2)
C117—H11C	0.98	C222—C223	1.385 (3)
C121—C126	1.396 (3)	C222—H222	0.95
C121—C122	1.398 (3)	C223—C224	1.395 (3)
C121—P1	1.825 (2)	C223—H223	0.95
C122—C123	1.396 (3)	C224—C225	1.397 (3)
C122—H122	0.95	C224—C227	1.507 (3)
C123—C124	1.389 (3)	C225—C226	1.386 (3)
C123—H123	0.95	C225—H225	0.95
C124—C125	1.393 (3)	C226—H226	0.95
C124—C127	1.512 (3)	C227—H22A	0.98
C125—C126	1.393 (3)	C227—H22B	0.98
C125—H125	0.95	C227—H22C	0.98
C126—H126	0.95	C231—C232	1.394 (3)
C127—H12A	0.98	C231—C236	1.398 (3)
C127—H12B	0.98	C231—P2	1.820 (2)
C127—H12C	0.98	C232—C233	1.392 (3)
C131—C132	1.397 (3)	C232—H232	0.95
C131—C136	1.401 (3)	C233—C234	1.391 (3)
C131—P1	1.825 (2)	C233—H233	0.95
C132—C133	1.389 (3)	C234—C235	1.397 (3)
C132—H132	0.95	C234—C237	1.509 (3)
C133—C134	1.397 (3)	C235—C236	1.386 (3)
C133—H133	0.95	C235—H235	0.95
C134—C135	1.395 (3)	C236—H236	0.95
C134—C137	1.507 (3)	C237—H23A	0.98
C135—C136	1.391 (3)	C237—H23B	0.98
C135—H135	0.95	C237—H23C	0.98
C136—H136	0.95	N—Ag	2.3519 (18)
C137—H13A	0.98	S—Ag^i^	2.6062 (6)
C137—H13B	0.98	Ag—P1	2.4516 (5)
C137—H13C	0.98	Ag—P2	2.4987 (5)
C211—C212	1.382 (3)	Ag—S^i^	2.6062 (6)
C211—C216	1.392 (3)		
			
N—C—S	178.0 (2)	C213—C214—C217	121.2 (2)
C116—C111—C112	118.43 (18)	C215—C214—C217	120.8 (2)
C116—C111—P1	123.51 (16)	C214—C215—C216	121.3 (2)
C112—C111—P1	117.43 (15)	C214—C215—H215	119.3
C113—C112—C111	120.79 (19)	C216—C215—H215	119.3
C113—C112—H112	119.6	C211—C216—C215	120.0 (2)
C111—C112—H112	119.6	C211—C216—H216	120
C112—C113—C114	121.0 (2)	C215—C216—H216	120
C112—C113—H113	119.5	C214—C217—H21A	109.5
C114—C113—H113	119.5	C214—C217—H21B	109.5
C115—C114—C113	118.09 (19)	H21A—C217—H21B	109.5
C115—C114—C117	122.0 (2)	C214—C217—H21C	109.5
C113—C114—C117	119.9 (2)	H21A—C217—H21C	109.5
C114—C115—C116	121.5 (2)	H21B—C217—H21C	109.5
C114—C115—H115	119.3	C226—C221—C222	118.1 (2)
C116—C115—H115	119.3	C226—C221—P2	118.37 (15)
C115—C116—C111	120.2 (2)	C222—C221—P2	123.54 (16)
C115—C116—H116	119.9	C223—C222—C221	120.5 (2)
C111—C116—H116	119.9	C223—C222—H222	119.8
C114—C117—H11A	109.5	C221—C222—H222	119.8
C114—C117—H11B	109.5	C222—C223—C224	121.5 (2)
H11A—C117—H11B	109.5	C222—C223—H223	119.3
C114—C117—H11C	109.5	C224—C223—H223	119.3
H11A—C117—H11C	109.5	C223—C224—C225	117.9 (2)
H11B—C117—H11C	109.5	C223—C224—C227	122.0 (2)
C126—C121—C122	118.87 (18)	C225—C224—C227	120.1 (2)
C126—C121—P1	117.34 (15)	C226—C225—C224	121.0 (2)
C122—C121—P1	123.70 (15)	C226—C225—H225	119.5
C123—C122—C121	120.1 (2)	C224—C225—H225	119.5
C123—C122—H122	120	C225—C226—C221	121.07 (19)
C121—C122—H122	120	C225—C226—H226	119.5
C124—C123—C122	121.2 (2)	C221—C226—H226	119.5
C124—C123—H123	119.4	C224—C227—H22A	109.5
C122—C123—H123	119.4	C224—C227—H22B	109.5
C123—C124—C125	118.55 (19)	H22A—C227—H22B	109.5
C123—C124—C127	121.7 (2)	C224—C227—H22C	109.5
C125—C124—C127	119.8 (2)	H22A—C227—H22C	109.5
C124—C125—C126	120.8 (2)	H22B—C227—H22C	109.5
C124—C125—H125	119.6	C232—C231—C236	118.39 (19)
C126—C125—H125	119.6	C232—C231—P2	124.08 (16)
C125—C126—C121	120.49 (19)	C236—C231—P2	117.53 (16)
C125—C126—H126	119.8	C233—C232—C231	120.4 (2)
C121—C126—H126	119.8	C233—C232—H232	119.8
C124—C127—H12A	109.5	C231—C232—H232	119.8
C124—C127—H12B	109.5	C232—C233—C234	121.4 (2)
H12A—C127—H12B	109.5	C232—C233—H233	119.3
C124—C127—H12C	109.5	C234—C233—H233	119.3
H12A—C127—H12C	109.5	C233—C234—C235	118.0 (2)
H12B—C127—H12C	109.5	C233—C234—C237	121.4 (2)
C132—C131—C136	118.36 (19)	C235—C234—C237	120.6 (2)
C132—C131—P1	122.71 (15)	C236—C235—C234	120.9 (2)
C136—C131—P1	118.84 (15)	C236—C235—H235	119.5
C133—C132—C131	120.69 (19)	C234—C235—H235	119.5
C133—C132—H132	119.7	C235—C236—C231	120.9 (2)
C131—C132—H132	119.7	C235—C236—H236	119.6
C132—C133—C134	121.21 (19)	C231—C236—H236	119.6
C132—C133—H133	119.4	C234—C237—H23A	109.5
C134—C133—H133	119.4	C234—C237—H23B	109.5
C133—C134—C135	118.0 (2)	H23A—C237—H23B	109.5
C133—C134—C137	121.1 (2)	C234—C237—H23C	109.5
C135—C134—C137	120.9 (2)	H23A—C237—H23C	109.5
C136—C135—C134	121.3 (2)	H23B—C237—H23C	109.5
C136—C135—H135	119.4	C—N—Ag	142.81 (16)
C134—C135—H135	119.4	C—S—Ag^i^	97.85 (7)
C135—C136—C131	120.51 (19)	N—Ag—P1	115.23 (5)
C135—C136—H136	119.7	N—Ag—P2	91.90 (5)
C131—C136—H136	119.7	P1—Ag—P2	119.826 (18)
C134—C137—H13A	109.5	N—Ag—S^i^	105.61 (5)
C134—C137—H13B	109.5	P1—Ag—S^i^	111.461 (18)
H13A—C137—H13B	109.5	P2—Ag—S^i^	110.655 (19)
C134—C137—H13C	109.5	C111—P1—C121	104.99 (9)
H13A—C137—H13C	109.5	C111—P1—C131	105.85 (9)
H13B—C137—H13C	109.5	C121—P1—C131	102.91 (9)
C212—C211—C216	118.4 (2)	C111—P1—Ag	110.53 (6)
C212—C211—P2	117.33 (17)	C121—P1—Ag	114.81 (7)
C216—C211—P2	124.24 (18)	C131—P1—Ag	116.68 (7)
C211—C212—C213	120.9 (2)	C231—P2—C211	103.99 (10)
C211—C212—H212	119.5	C231—P2—C221	105.56 (9)
C213—C212—H212	119.5	C211—P2—C221	102.21 (11)
C214—C213—C212	121.2 (2)	C231—P2—Ag	111.11 (7)
C214—C213—H213	119.4	C211—P2—Ag	117.23 (8)
C212—C213—H213	119.4	C221—P2—Ag	115.42 (7)
C213—C214—C215	117.99 (19)		
			
C116—C111—C112—C113	1.2 (3)	P2—C231—C236—C235	179.95 (16)
P1—C111—C112—C113	−170.13 (16)	C—N—Ag—P1	175.2 (3)
C111—C112—C113—C114	0.5 (3)	C—N—Ag—P2	−60.3 (3)
C112—C113—C114—C115	−1.7 (3)	C—N—Ag—S^i^	51.7 (3)
C112—C113—C114—C117	176.8 (2)	C116—C111—P1—C121	122.96 (17)
C113—C114—C115—C116	1.4 (3)	C112—C111—P1—C121	−66.24 (17)
C117—C114—C115—C116	−177.1 (2)	C116—C111—P1—C131	14.51 (19)
C114—C115—C116—C111	0.3 (3)	C112—C111—P1—C131	−174.69 (15)
C112—C111—C116—C115	−1.5 (3)	C116—C111—P1—Ag	−112.70 (16)
P1—C111—C116—C115	169.17 (16)	C112—C111—P1—Ag	58.10 (16)
C126—C121—C122—C123	1.3 (3)	C126—C121—P1—C111	160.41 (16)
P1—C121—C122—C123	−175.01 (16)	C122—C121—P1—C111	−23.2 (2)
C121—C122—C123—C124	−0.1 (3)	C126—C121—P1—C131	−89.01 (17)
C122—C123—C124—C125	−1.1 (3)	C122—C121—P1—C131	87.39 (19)
C122—C123—C124—C127	178.7 (2)	C126—C121—P1—Ag	38.83 (18)
C123—C124—C125—C126	1.1 (3)	C122—C121—P1—Ag	−144.77 (16)
C127—C124—C125—C126	−178.7 (2)	C132—C131—P1—C111	84.96 (19)
C124—C125—C126—C121	0.1 (3)	C136—C131—P1—C111	−98.78 (17)
C122—C121—C126—C125	−1.4 (3)	C132—C131—P1—C121	−25.0 (2)
P1—C121—C126—C125	175.23 (17)	C136—C131—P1—C121	151.28 (17)
C136—C131—C132—C133	−0.8 (3)	C132—C131—P1—Ag	−151.62 (15)
P1—C131—C132—C133	175.47 (17)	C136—C131—P1—Ag	24.63 (19)
C131—C132—C133—C134	−0.1 (3)	N—Ag—P1—C111	104.30 (9)
C132—C133—C134—C135	1.4 (3)	P2—Ag—P1—C111	−3.90 (7)
C132—C133—C134—C137	−177.3 (2)	S^i^—Ag—P1—C111	−135.37 (7)
C133—C134—C135—C136	−2.0 (3)	N—Ag—P1—C121	−137.18 (9)
C137—C134—C135—C136	176.7 (2)	P2—Ag—P1—C121	114.62 (7)
C134—C135—C136—C131	1.1 (3)	S^i^—Ag—P1—C121	−16.86 (8)
C132—C131—C136—C135	0.3 (3)	N—Ag—P1—C131	−16.66 (9)
P1—C131—C136—C135	−176.14 (17)	P2—Ag—P1—C131	−124.87 (7)
C216—C211—C212—C213	−3.8 (4)	S^i^—Ag—P1—C131	103.66 (7)
P2—C211—C212—C213	175.1 (2)	C232—C231—P2—C211	84.7 (2)
C211—C212—C213—C214	1.2 (4)	C236—C231—P2—C211	−95.14 (18)
C212—C213—C214—C215	1.8 (4)	C232—C231—P2—C221	−22.5 (2)
C212—C213—C214—C217	−177.5 (2)	C236—C231—P2—C221	157.67 (16)
C213—C214—C215—C216	−2.2 (4)	C232—C231—P2—Ag	−148.33 (16)
C217—C214—C215—C216	177.1 (2)	C236—C231—P2—Ag	31.86 (17)
C212—C211—C216—C215	3.4 (4)	C212—C211—P2—C231	167.9 (2)
P2—C211—C216—C215	−175.4 (2)	C216—C211—P2—C231	−13.3 (3)
C214—C215—C216—C211	−0.4 (4)	C212—C211—P2—C221	−82.4 (2)
C226—C221—C222—C223	1.0 (3)	C216—C211—P2—C221	96.4 (3)
P2—C221—C222—C223	179.46 (17)	C212—C211—P2—Ag	44.8 (3)
C221—C222—C223—C224	−0.2 (3)	C216—C211—P2—Ag	−136.4 (2)
C222—C223—C224—C225	−0.4 (3)	C226—C221—P2—C231	−105.37 (17)
C222—C223—C224—C227	179.2 (2)	C222—C221—P2—C231	76.19 (19)
C223—C224—C225—C226	0.1 (3)	C226—C221—P2—C211	146.15 (17)
C227—C224—C225—C226	−179.4 (2)	C222—C221—P2—C211	−32.3 (2)
C224—C225—C226—C221	0.7 (3)	C226—C221—P2—Ag	17.74 (18)
C222—C221—C226—C225	−1.2 (3)	C222—C221—P2—Ag	−160.69 (16)
P2—C221—C226—C225	−179.76 (16)	N—Ag—P2—C231	−84.01 (8)
C236—C231—C232—C233	0.6 (3)	P1—Ag—P2—C231	36.69 (7)
P2—C231—C232—C233	−179.18 (16)	S^i^—Ag—P2—C231	168.51 (7)
C231—C232—C233—C234	−1.1 (3)	N—Ag—P2—C211	35.34 (11)
C232—C233—C234—C235	0.8 (3)	P1—Ag—P2—C211	156.04 (10)
C232—C233—C234—C237	−179.3 (2)	S^i^—Ag—P2—C211	−72.14 (10)
C233—C234—C235—C236	0.0 (3)	N—Ag—P2—C221	155.87 (9)
C237—C234—C235—C236	−180.0 (2)	P1—Ag—P2—C221	−83.43 (7)
C234—C235—C236—C231	−0.4 (3)	S^i^—Ag—P2—C221	48.39 (7)
C232—C231—C236—C235	0.1 (3)		

Symmetry codes: (i) −*x*+1, −*y*+1, −*z*+1.

### Hydrogen-bond geometry (Å, °)

**Table:**

Cg1 and Cg6 are the centroids of the C111–C116 and C231–C236 benzene rings, respectively.

**Table:**

*D*—H···*A*	*D*—H	H···*A*	*D*···*A*	*D*—H···*A*
C135—H135···Cg6^ii^	0.95	2.86	3.772 (2)	161
C225—H225···Cg1^iii^	0.95	2.73	3.568 (2)	147

Symmetry codes: (ii) −*x*, −*y*+1, −*z*+1; (iii) −*x*, −*y*+1, −*z*.
